# The coronary microcirculation in sepsis: not of micro-importance

**DOI:** 10.21542/gcsp.2020.30

**Published:** 2020-12-31

**Authors:** Alessia Argirò, Iacopo Olivotto

**Affiliations:** 1 Cardiomyopathy Unit, Careggi University Hospital, Florence, Italy

The coronary arterial system consists of a continuous network of functionally distinct vessels of decreasing size.^1^ The epicardial arteries (>500 *μ*m) have primarily a conductance function and, therefore, physiologically offer minimal resistance to flow. The microcirculation, including pre-arterioles (100 to 500 *μ*m) and arterioles (<100 *μ*m), on the other hand, is the main active determinant of resistance within the coronary tree, responsible for the metabolic regulation of regional blood flow to the myocardium.^1,2^

**Figure 1.**
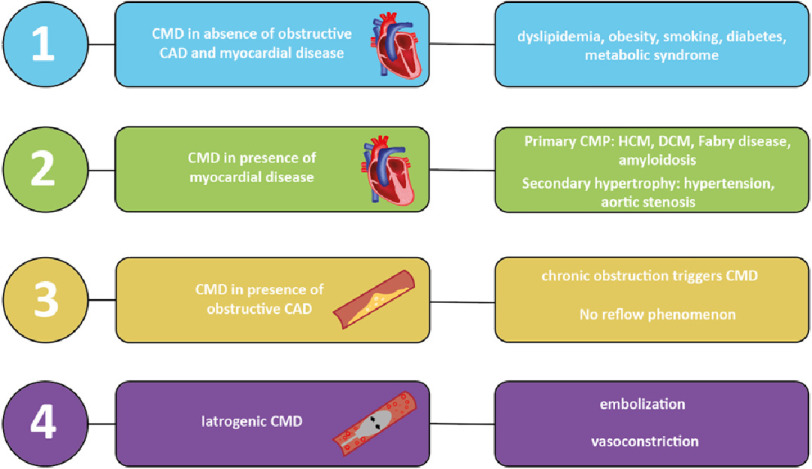
Classification of coronary microvascular dysfunction. CMD: coronary microvascular dysfunction. CAD: coronary artery disease. CMP: cardiomyopathies. HCM: hypertrophic cardiomyopathy. DCM: dilated cardiomyopathy. **Group 1**: Metabolic dysregulation due to dyslipidemia, obesity, smoking, diabetes and metabolic syndrome may impair microvascular structure and endothelium dependent and independent vascular function.^18^ **Group 2**: CMD has been reported in both primary cardiomyopathies (hypertrophic cardiomyopathy, dilated cardiomyopathy, Anderson-Fabry disease and amyloidosis) and secondary hypertrophy settings (aortic stenosis and hypertension).^19–21^ **Group 3**: Chronic coronary obstruction can trigger CMD that may persist even after successful revascularization.^22,23^ In revascularized acute coronary syndromes CMD may be responsible for suboptimal reperfusion (i.e., “no reflow” phenomenon^24^), caused by endothelial injury and/or distal embolization and is associated with adverse cardiovascular events in STEMI patients.^25^ **Group 4**: After percutaneous coronary intervention, vasoconstriction due to epicardial and arteriolar alfa adrenergic receptors activation^26^ and embolization of plaque deriving material in the microcirculation may cause microinfarcts affecting long term clinical outcome.^27^

Structural and/or functional abnormalities in coronary microcirculation (i.e., coronary microvascular dysfunction, CMD), occurring in a wide spectrum of cardiovascular diseases, are classifiable into four groups^1^ (Figure 1):

(1) Coronary microvascular dysfunction in the absence of obstructive coronary artery disease (CAD) and myocardial diseases,
(2) Coronary microvascular dysfunction in the presence of myocardial diseases,
(3) Coronary microvascular dysfunction in the presence of obstructive CAD,
(4) Iatrogenic coronary microvascular dysfunction.

CMD is an intricate and often key component of heart pathophysiology. It arises from changes in both microvascular function and structure and is strongly associated with endothelial dysfunction. CMD is highly prevalent and determines the fate of numerous chronic heart diseases, but may be pivotal also in acute conditions such as acute myocardial infarction and various types of shock. Hence, the growing necessity of a deeper understanding of microcirculatory pathophysiology and developing effective therapeutic interventions.

Sepsis is a life-threatening organ dysfunction caused by a dysregulated host response to infection^3^, and a leading cause of intensive care unit morbidity and mortality.^4^ Cardiac involvement in septic patients is associated with severe outcome ^5,6^ and coronary microcirculation is of paramount importance in this setting. CMD due to inflammatory dysregulation is part of the wider spectrum of CMD and, although not included in the original classification, appears classifiable as a type 1 dysfunction. In septic patients, endothelial proinflammatory activation increases endothelial permeability leading to myocardial oedema, which has been reported in both experimental and clinical sepsis. ^7,8^ Acute myocardial oedema has severe pathophysiological consequences including systolic^9^ and diastolic dysfunction, in both active (cross bridge detachment) and passive (myocardial stiffness) components.^10,11^ Such impairment continues even after the resolution of the oedema.^12^ Furthermore, inflammatory stimuli upregulate cell adhesion molecules and enhance leucocyte adhesion and activity exposing the myocardium to contractile depressant factors (e.g., TNF alfa and IL-1) and reactive oxygen species.^13^

In a recent issue of the journal, McBride et. al^14^ provide important insights into sepsis-related microvascular dysfunction, by reviewing its peculiar pathogenesis and features as compared to dengue fever-associated shock. As reviewed by the Authors, septic shock and dengue share common pathophysiological features including reduced endothelial (nitric oxide) dependent vasodilation, endothelial and immune system cell activation, glycocalyx shedding and plasma leakage through slack endothelial junctions. The latter phenomenon, however, is exaggerated in dengue shock, while microvascular tone impairment appears less marked compared to septic shock. The reasons behind the peculiar behaviour and specific pattern of dengue shock remain unresolved and may involve the degradation of glycocalyx components mediated by viral glycoprotein non-structural 1 (NS1) and the excessive host inflammatory response to dengue infection.

While largely unexplained, this pathophysiological difference has important management implications, implying a greater need for fluid-based rather than vasopressor-mediated approach in dengue shock, as opposed to classic septic shock.

Research into novel treatment for microvascular dysfunction remains an unmet clinical need both in the acute (sepsis, STEMI) and chronic (hypertensive heart disease, microvascular angina, cardiomyopathies) setting. Data on the use of platelet inhibitors are insufficiently established to provide clinical recommendations. Yet, clinical studies investigating the adenosine-mediated vasodilator effect of ticagrelor are ongoing.^15^ Furthermore ACE inhibitors and statins may counteract oxidative stress and may be benefit in patients with CMD, particularly when associated with the classic cardiovascular risk factors.^16^ In other contexts such as cardiomyopathies, however, promising preclinical studies have failed to translate into successful clinical results.^17^ Further research is urgently warranted on this anatomically micro, clinically macro, problem.
